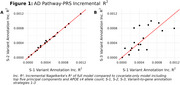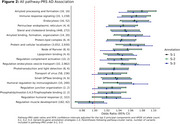# Evaluation of variant‐to‐gene annotation strategies in Alzheimer's Disease pathway‐specific polygenic risk score construction

**DOI:** 10.1002/alz70855_105686

**Published:** 2025-12-24

**Authors:** Katrina Bazemore, Taha Iqbal, Jin Sha, Joseph Manuel, Jacob Haut, Wan‐Ping Lee, Yi Zhao, Otto Valladares, Cornelia M Van Duijn, Li‐San Wang, Gerald D. Schellenberg, Jin Jin, Adam C. Naj

**Affiliations:** ^1^ University of Pennsylvania, Perelman School of Medicine, Philadelphia, PA, USA; ^2^ Penn Neurodegeneration Genomics Center, University of Pennsylvania, Perelman School of Medicine, Philadelphia, PA, USA; ^3^ University of Pennsylvania Perelman School of Medicine, Philadelphia, PA, USA; ^4^ Institute for Biomedical Informatics, Perelman School of Medicine, University of Pennsylvania, Philadelphia, PA, USA; ^5^ University of Oxford, Oxford, Oxfordshire, United Kingdom; ^6^ Penn Neurodegeneration Genomics Center, Perelman School of Medicine, University of Pennsylvania, Philadelphia, PA, USA; ^7^ Department of Biostatistics, Epidemiology and Informatics, University of Pennsylvania Perelman School of Medicine, Philadelphia, PA, USA

## Abstract

**Background:**

Pathway‐specific polygenic risk scores (pathway‐PRS) are a recently developed tool to measure genetic susceptibility to complex diseases along specific biological pathways. Positional strategies annotating risk variants to pathways may not capture genetic susceptibility in non‐coding regions. Risk variant localization to non‐coding regions is characteristic of many complex diseases, including Alzheimer's Disease (AD). We evaluated the impact of three annotation strategies on AD pathway‐PRS performance in the UK Biobank (UKB).

**Methods:**

We performed pathway enrichment analysis on Kunkle et al (*Nat Genet* 2019) summary statistics. Pathways meeting an adjusted *p*‐value threshold were clustered based on overlapping gene content. Variants were annotated to pathway‐cluster genes based on variant position within 35kb upstream to 10kb downstream of gene boundaries (“S‐1”); S‐1 adding chromatin interaction and expression quantitative trait loci (eQTL)‐based annotations on prioritized variants (“S‐2”); and variant position within gene exon or promoter regions along with genome‐wide chromatin interaction and eQTL‐based annotations (“S‐3”). Variants annotated to pathway‐cluster genes were included in pathway‐PRS following clumping and thresholding, with tuning in an independent UKB training set (true/proxy cases=33,370; controls=229,486) and validation in a UKB testing set (true/proxy cases=8,309; controls=57,516).

**Results:**

We identified 20 pathway‐clusters representing 37 Gene Ontology (GO) pathways meeting adjusted *p*≤0.25. S‐2 annotated 0.5% more and S‐3 56% fewer variants to pathway‐cluster genes compared to S‐1. There was little change in odds‐ratios (OR) and incremental R^2^ (Inc.R^2^) between S‐1 and S‐2. Larger differences were observed between S‐3 and S‐1, with both increases and decreases in OR and Inc.R^2^ (Figures 1 & 2). The pathway‐PRS with the largest OR under S‐1 and S‐2 (OR=1.071, *p* = 4.5×10^‐09^) contained four pathways related to regulation of amide metabolism (GO: 0034249), amyloid‐beta formation (GO: 1902430), and amyloid precursor catabolism (GO: 1902991 and GO: 1902992). The pathway‐PRS with the largest OR under S‐3 (OR=1.073, *p* = 2.7×10^‐09^) contained four protein and cellular localization pathways (GO:0060341, GO:2000009, GO:0070201, and GO:0032880) (Figure 2).

**Conclusion:**

The inclusion of regulatory variants in AD pathway‐PRS lead to the prioritization of protein and cellular localization pathways over amyloid pathways. Additional strategies for pathway‐PRS construction, such as incorporating functional annotations as priors, will be tested in future work.